# Alignment effects in beer mugs: Automatic action activation or response competition?

**DOI:** 10.3758/s13414-016-1130-7

**Published:** 2016-05-16

**Authors:** Sander A. Roest, Diane Pecher, Lilian Naeije, René Zeelenberg

**Affiliations:** Department of Psychology, Erasmus University Rotterdam, Woudestein T13-50, P.O. Box 1738, 3000 DR Rotterdam, The Netherlands

**Keywords:** Simon effect, Spatial alignment, Stimulus response compatibility, Go/no-go task, Choice-reaction task

## Abstract

Responses to objects with a graspable handle are faster when the response hand and handle orientation are aligned (e.g., a key press with the right hand is required and the object handle is oriented to the right) than when they are not aligned. This effect could be explained by automatic activation of specific motor programs when an object is viewed. Alternatively, the effect could be explained by competition at the response level. Participants performed a reach-and-grasp or reach-and-button-press action with their left or right hand in response to the color of a beer mug. The alignment effect did not vary as a function of the type of action. In addition, the alignment effect disappeared in a go/no-go version of the task. The same results were obtained when participants made upright/inverted decisions, so that object shape was task-relevant. Our results indicate that alignment effects are not due to automatic motor activation of the left or right limb.

Recent studies suggest that pictures of objects potentiate motor actions that are compatible with the grasping actions that would be performed on a real object. For example, responses to stimulus properties, such as color or upright/inverted orientation, are faster if the response hand is on the same side as the object’s graspable part than if it is on the other side (Tucker & Ellis, 1998; Tipper, Paul, & Hayes, 2006). The present study examined whether such lateralized grasping responses are activated automatically by pictures of objects^1^ (Goslin, Dixon, Fischer, Cangelosi, & Ellis, 2012; Handy, Grafton, Schroff, Ketay, & Gazzaniga, 2003; Iani, Baroni, Pellicano, & Nicoletti, 2011; Makris, Hadar, & Yarrow, 2011; Pellicano, Iani, Borghi, Rubichi, & Nicoletti, 2010; Tucker & Ellis, 1998) or occur because of task-related response competition (Bub & Masson, 2010) or abstract spatial coding (Cho & Proctor, 2010, 2011; Lien, Gray, Jardin, & Proctor, 2014; Phillips & Ward, 2002; Song, Chen, & Proctor, 2014).

One of the first studies investigating whether object pictures automatically activate lateralized responses was performed by Tucker and Ellis (1998). Participants responded to upright or inverted graspable objects (e.g., a frying pan or teapot). The object’s handle was oriented to either the left or the right. Participants responded with a left or right key press to the object’s orientation (upright or inverted). They responded faster when their response hand was aligned with the orientation of the handle (e.g., the correct response was with the right hand and the object handle was oriented to the right side) compared to when their response hand was misaligned with the handle (e.g., the correct response was with the right hand and the object handle was oriented to the left side). This *alignment effect* was found when participants had to respond with either their right hand or their left hand (between-hands condition) but not when participants had to respond with either their index (left) or their middle (right) finger of the same hand (within-hand condition). Tucker and Ellis attributed the alignment effect to an automatic lateralized grasping response: The object handle automatically activates a specific motor response of the aligned hand.

Other studies, however, have questioned this explanation of alignment effects (e.g., Cho & Proctor, 2010, 2011, 2013; Lien et al., 2014; Phillips & Ward, 2002; Proctor & Miles, 2014; Song et al., 2014). According to some of these researchers, alignment effects can be explained by relative spatial coding for the left or right response. On this account, performance is better when spatial dimensions of the stimulus and response correspond than when they do not. Most important, these spatial codes are abstract and are independent of the specifics of the motor response. According to this view, alignment effects are in fact variations of the Simon effect (Simon, 1969). The dimensional overlap (DO) model (Kornblum, Hasbroucq, & Osman, 1990; Zhang, Zhang, & Kornblum, 1999) and the theory of event coding (TEC; Hommel, 2009; Memelink & Hommel, 2013) argue that Simon effects occur because of overlap in stimulus and response dimensions. According to TEC, stimuli and responses are coded in the same way and use the same stimulus maps. In a Simon task, this causes a delayed response if the primed stimulus map does not correspond with the required response. On this account, alignment effects should occur when responses correspond spatially with the spatial stimulus dimension but should not depend on overlap between other aspects of the response and the motor actions associated to grasping the depicted object. An important aspect of the Simon effect explanation is that alignment effects should also occur when responses are made by the left or right finger within the same hand. As reported earlier, this had not been found by Tucker and Ellis (1998), which led them to conclude that the alignment effect is due to an automatic lateralized grasping response. Cho and Proctor (2010, 2011), however, did not replicate these findings. That is, they did find an alignment effect when participants made within-hand responses. In addition, another study obtained alignment effects when participants responded with their feet (Phillips & Ward, 2002). Other studies have shown alignment effects for pictures of objects that have no graspable handle, such as clock faces (Anderson, Yamagishi, & Karavia, 2002) or animals (Matheson, White, & McMullen, 2014). These findings all argue against automatic activation of grasping responses as an explanation and suggest that the alignment effect might be better explained by abstract spatial coding of responses for the left or right.

A third explanation of the alignment effect is that the effect is due to activation of competing motor actions, whose activation depends on specific task requirements (Bub & Masson, 2010; Tipper et al., 2006; Yu, Abrams, & Zacks, 2014). Task requirements could direct attention to a particular attribute of an object or influence the action intention of the participant, which in turn will influence the likelihood that grasping responses are activated. In the study by Tucker and Ellis (1998), described earlier, participants had to respond to the orientation (upright or inverted) of the object. In this task the object’s shape needed to be attended in order to respond correctly. To investigate the importance of attention to shape, Tipper et al. (2006) had participants look at left- or right-oriented door handles that varied in shape and color. They found an alignment effect when participants had to respond with the left or right hand in response to the shape of the handles but not if they had to respond to their color. These findings suggest that attending to the shape of an object is crucial to automatically activate the specific lateralized motor actions of the left or right hand. In other words, there should be no alignment effect when the shape of the object is irrelevant to the response.

Bub and Masson (2010) argued, however, that key-press responses might not be sufficient to activate the specific lateralized motor actions of the left or right hand when the object’s shape is irrelevant to the response. Rather, they argue, only when the task requires goal-directed actions (such as a reach action) will alignment of the object handle with the response hand facilitate responses. They further suggest that potential actions for each limb are initially activated simultaneously and compete for activation. Activation of a lateralized motor action thus evolves over time and depends on task demands. In Bub and Masson’s experiments, participants viewed beer mugs with their handles oriented either to the left or to the right. Participants responded to the color of the beer mug (red or blue) with their left or right hand. Instead of a button press, Bub and Masson used a response element (a C-shaped handle) that participants had to grasp. They also used two types of dependent measures, lift-off time (measured as the time from the onset of the relevant stimulus to the beginning of the movement) and movement time (measured as the time from lift-off to grasping of the response device). They obtained an alignment effect in lift-off time which emerged over time, becoming larger when there was a delay (195 ms to 495 ms) between the onset of the object and the onset of the object color. When participants were asked to respond with left or right key presses instead (Experiment 2), rather than reach and grasp actions, no alignment effects were found, replicating the earlier findings of Tipper et al. (2006). Bub and Masson argued that left/right hand selection and a reaching action are required to show alignment effects when the object’s shape is irrelevant to the response. This hand selection builds up over time, and alignment effects are revealed only when participants are required to perform actions that depend on specific motor goals.

Thus, alignment effects could be due to automatically activated grasping responses, to task-induced factors such as relative spatial coding of responses (e.g., Proctor & Miles, 2014), or to activation of and competition between lateralized responses (Bub & Masson, 2010). These three explanations make different predictions regarding the role of action similarity and competition between responses. First, if the alignment effect is due to automatic activation of a grasping response, it should be sensitive to the overlap between the motor action evoked by the object picture and the motor action required to make a response. Bub and Masson (2010, Experiment 1) found alignment effects in lift-off time when participants had to make a reach-and-grasp response, even when the grasp response itself did not match the orientation of the grasp implied by the object^2^ (e.g., a horizontal cylinder grasp response to a stimulus object with a vertical handle). With just a key-press response (i.e., no reach action was performed), however, the alignment effect was absent. These conditions, however, differed not only in whether the response involved a reach and grasp action or a simple key press but also in the point in time of the response action at which RT was measured. For the reach-and-grasp conditions, the alignment effect was obtained in the lift-off time, that is, the start of the response. For the key-press responses, however, the RT was measured at the moment of the key press, that is, the endpoint of the response. As Bub and Masson (2010) speculated, an alignment effect might occur if the key press had to be made after a reach action. Similar alignment effects for reach-and-grasp and reach-and-key-press responses would indicate, however, that the effect is not due to automatic activation of grasping actions but rather to competition at the response level.

Whereas the congruent and incongruent grasps used by Bub and Masson (2010) might still be considered similar in terms of hand configuration (i.e., they both required a full hand grip), a key-press response requires a different hand configuration than a grasp. In Experiment 1, we investigated whether the alignment effect is larger in conditions where participants perform reach-and-grasp actions than in conditions where they perform reach actions without grasping.

A second prediction that follows from the view that grasping actions are activated automatically is that alignment effects should occur even when there is no need to spatially code responses or to resolve competition between responses. If a motor action is activated by an object, it should influence response actions such that performance is better for compatible actions than for incompatible actions. This prediction was investigated in Experiments 2 and 3.

## Experiment 1

In Experiment 1, we aimed to replicate and extend the findings of Bub and Masson (2010, Experiment 1) and confirm their predictions regarding reach-without-grasp responses. In their experiments, participants responded to the color of a beer mug with a reach-and-grasp response (Experiment 1) or a key press (Experiment 2). For reach-and-grasp responses, alignment effects in lift-off time were found. No alignment effect was obtained for the key-press condition (Experiment 2) in which participants simply pressed a key on the keyboard instead of making a reach-and-grasp movement. Bub and Masson (2010) noted that simple button-press responses might not be an optimal comparison for reach-and-grasp responses. A reach-and-grasp action consists of different phases (lift-off and movement) while a simple button press does not result in a release or a movement of the entire hand. To better compare a grasp with a press response, we used a condition where participants reached for a button (in our case, the top of a small, round screw, which participants had to touch, henceforth referred to as button press). The response action (cylinder grasp or button press) was manipulated between participants, whereas the response side was manipulated within participants. Thus, in our experiment, competition occurred between left and right responses but not between grasping and pressing. If the alignment effect is due to competition between responses or abstract spatial coding, an alignment effect should be observed for both grasping and pressing actions. If, on the other hand, the alignment effect is due to automatically activated grasping actions toward the pictured object, the effect should be larger in the cylinder-grasp condition than in the button-press condition.

### Method

#### Participants

Sixty psychology students at Erasmus University Rotterdam participated in the experiment. They participated either voluntarily (i.e., without compensation) or for course credit. Participants were randomly assigned to one of two groups; 30 were assigned to the cylinder-grasp group, which was asked to reach and grasp a metal cylinder as a response (cylinder-grasp condition), and 30 were assigned to the button-press group, which was asked to reach and touch a small metal screw as a response (button-press condition). Four additional participants were tested, but their data were not included in the analyses; three participants were excluded because of hardware malfunctions, and one participant was excluded because of failing to perform the prescribed power grasp on every trial.

#### Materials and apparatus

The same stimuli were used in all experiments reported here. We used the original photographs of Bub and Masson (2010). These consisted of grayscale photographs of a handled beer mug and two colored versions (red and blue) of the same beer mug. Each picture had two versions: one with the handle oriented to the right and one with the handle oriented to the left. The image size of the beer mug on the computer screen was 5.0 cm vertically and 3.6 cm horizontally. When viewed at a 50-cm distance, this corresponded to visual angles of 5.7° vertically and 4.1° horizontally.

**Fig. 1:**
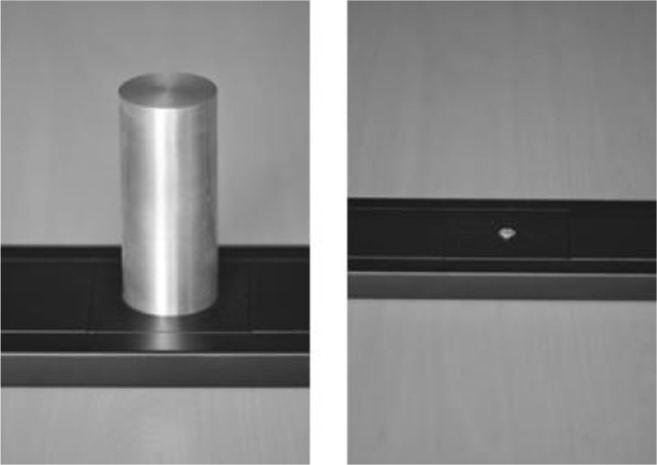
Close-up of the Grabbit response elements used in the present study (cylinder on the left and screw on the right)

Stimulus presentation and response collection were controlled by a computer programmed in E-Prime 2.0 (Psychology Software Tools, Pittsburgh, PA). As a response device we used the Grabbit, a modular system developed in our lab to measure reaction times. The Grabbit was modeled after the Graspasaurus (Bub, Masson, & Cree, 2008) and consists of an MDF board to which different response elements can be attached. In Experiment 1, two different response elements were used (in a between-subjects design): a metal cylinder with a height of 14 cm and a diameter of 6 cm that affords a vertical power grasp response, and a small round metal screw that affords a poke response (see Fig. 1). The response element triggered a signal when it was touched. In order for the response device to function participants, had to put a small electrode on their leg or foot, such that if they touched the response element, they closed an electric circuit, sending a signal to the computer via a Makey Makey (JoyLabz). For safety reasons, a galvanic isolation was placed between the Makey Makey and the computer.

**Fig. 2:**
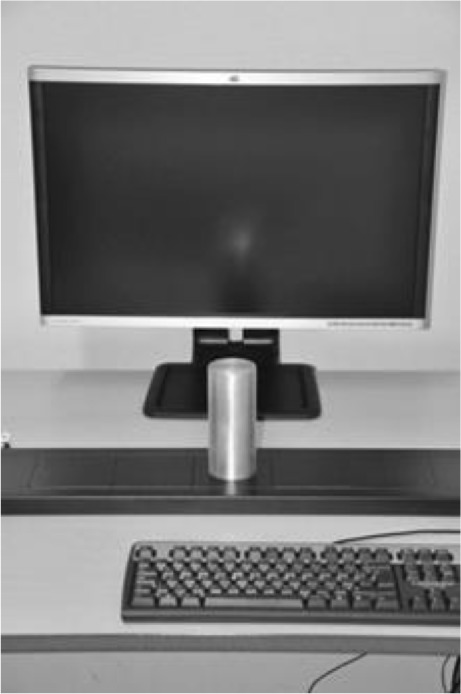
Setup with keyboard, Grabbit (with cylinder), and computer monitor

In addition to the Grabbit, a keyboard was used to hold down two keys in order to record lift-off times. The keyboard was placed with the F4 key on the midline between the response element and the participant so that the *z* and *m* keys were at the same distance away from the center of the response element. The general setup is shown in Fig. 2.

#### Procedure

The experiment consisted of a practice phase of 40 trials followed by six blocks of 60 critical trials each (360 critical trials in total). Each block consisted of 15 trials of each of the four combinations of handle orientation and response side, resulting in 30 aligned trials and 30 misaligned trials per block. Participants in the cylinder-grasp condition were asked to respond by grasping the cylinder in response to the color of the beer mug. Participants in the button-press condition were asked to touch the small screw in response to the color of the beer mug. The response device was located on the midline between the participant and the computer monitor.

In the practice phase of the cylinder-grasp condition, participants saw a picture of a hand forming a power grasp (identical to the one used by Bub & Masson, 2010) together with a rectangle colored red or blue. In the practice phase of the button-press condition, participants only saw the rectangle colored red or blue with instructions as to which hand they had to use to make a response. Red indicated a right-hand response and blue indicated a left-hand response for half of the participants in each group, and the reverse color-response side pairing was used for the other participants. Participants were instructed to press down the *z* and *m* keys on the keyboard with their left- and right-hand index fingers before the start of each trial (this instruction was given before practice trials began and did not appear on the screen between trials). After these instructions, participants performed 40 practice trials in which they had to respond to colored rectangles. Each trial started with the presentation of a fixation cross (*+*) for 1,500 ms. Participants responded to the color by moving the correct hand and grasping the cylinder or touching the screw on the response device (depending on the condition to which they had been assigned). Feedback was displayed (*incorrect hand*) for 1,500 ms when participants used the incorrect response hand. Each trial ended with an intertrial interval of 1,000 ms.

**Fig. 3:**
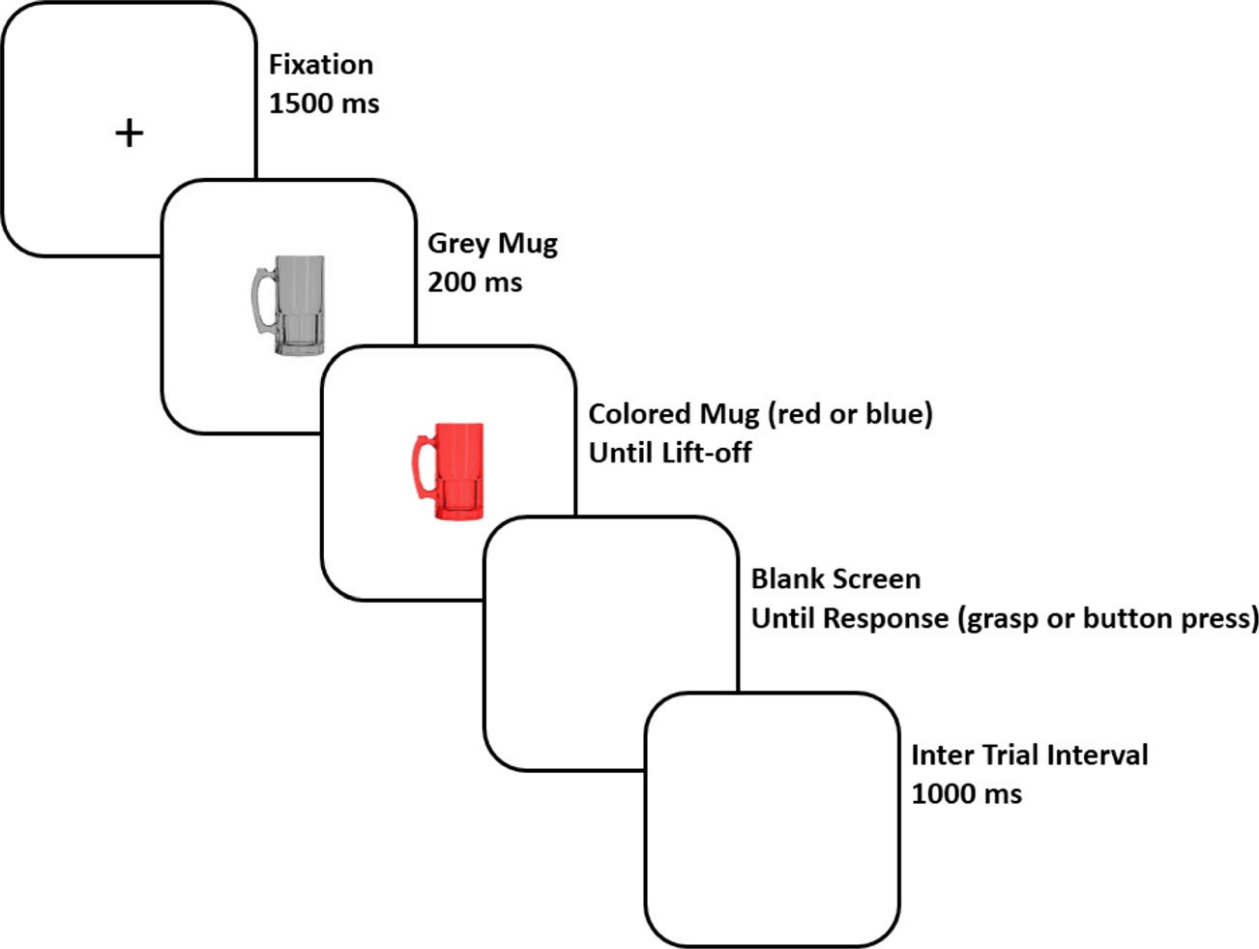
Example of the trial sequence used in Experiment 1. Prior to the start of the trial, participants pressed down the *z* and *m* keys on the keyboard. Participants responded by moving the left or right hand (depending on the color of the beer mug) to the Grabbit response element. This allowed us to measure both the lift-off time and the movement time. The type of response (cylinder grasp or button press) was manipulated between subjects. The colored mug remained visible until the *m* or *z* key was released

After these practice trials, participants were told they now had to respond to the color of the beer mug. The six blocks of critical trials followed these instructions. Each trial started with a fixation cross (*+*) presented for 1,500 ms. After the fixation cross, a photograph of a grayscale beer mug with the handle oriented to the left or to the right was shown. After a cue delay of 200 ms, the mug changed color to red or blue, indicating which response hand the participant had to use. When the *z* or *m* key was released, the colored beer mug immediately disappeared from the monitor; the monitor was blank while the hand moved to the response device. After the participant had grasped the cylinder or touched the screw, there was a 1,000 ms intertrial interval before the next trial started. During this interval, participants returned their fingers to the *z* and *m* keys on the keyboard. When participants used the incorrect response hand, feedback was displayed (*incorrect hand*) for 1,500 ms before the intertrial interval. After each block, participants could take a self-paced break. Participants were randomly assigned to either the cylinder-grasp or the button-press condition. Trials were presented in a different random order for each participant. An example of a single trial is shown in Fig. 3.

### Results and discussion

The mean accuracy across participants was 98% (range = 93%–100%). No participants were excluded based on accuracy. Following Bub and Masson (2010), lift-off times faster than 200 ms or slower than 1,000 ms and movement times slower than 800 ms were considered outliers and were excluded from the RT analyses. This resulted in the exclusion of 2.2% of correct responses in lift-off time and movement time. The same outlier criteria were used in Experiment 2. The raw data for Experiments 1–3 are available on the Open Science Framework (https://osf.io/fe6pj/).

Separate analyses were performed to assess the effects of the experimental manipulations on lift-off time (the time from the onset of the color on the computer screen until release of the *z* or *m* key on the keyboard) and movement time (the time to move the response hand from the keyboard to the Grabbit response element).

#### Lift-off time

**Fig. 4:**
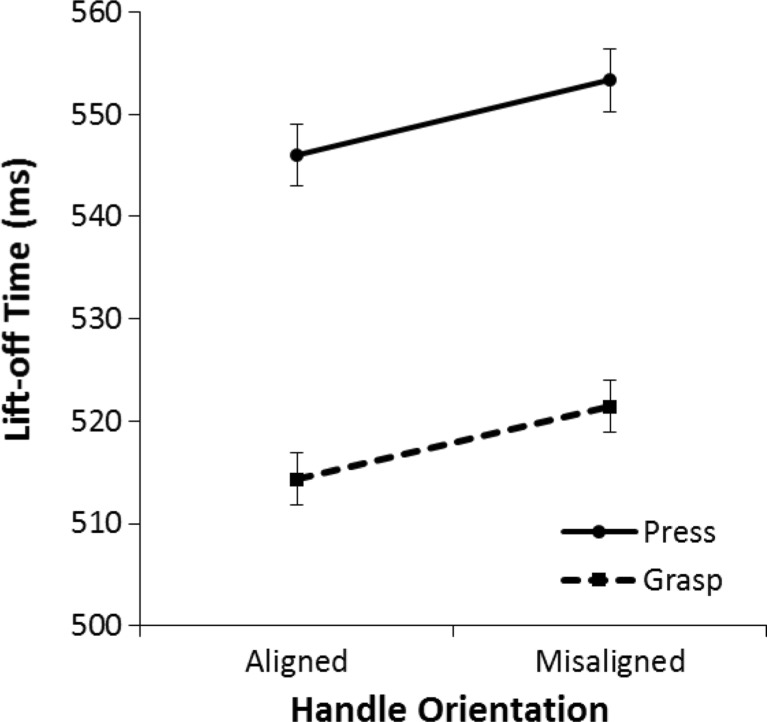
Mean lift-off times in Experiment 1 for the button-press group and the cylinder-grasp group. Lift-off time is the time it takes from color onset to the beginning of the movement (release of the keyboard). The error bars indicate the standard error of the mean based on the within-subjects difference between the aligned condition and the misaligned condition

Figure 4 displays the mean lift-off times for each condition. As can be seen, for both cylinder-grasp and button-press responses, lift-off times were faster when the handle of the beer mug was aligned with the correct response hand than when the handle of the beer mug was not aligned with the correct response hand. This conclusion was supported by a 2 (aligned vs. misaligned) × 2 (cylinder grasp vs. button press) mixed analysis of variance (ANOVA) on the mean lift-off time for correct trials. Lift-off times were faster on aligned trials than on misaligned trials, *F*(1, 58) = 13.03, *p* < .001, η^2^ = .18. There was a marginally significant main effect of response type; participants who performed a grasp response had faster lift-off times than participants who performed a button-press response, *F*(1, 58) = 3.93, *p* = .052, η^2^ = .06. There was no interaction between alignment (aligned vs. misaligned) and response type (cylinder grasp vs. button press), *F*(1, 58) = 0.004, *p* = .95, η^2^ < .001.

Even though the interaction was not significant, we performed post hoc repeated-measures ANOVAs to analyze alignment effects separately for each group. These analyses revealed that, for both the cylinder-grasp group and the button-press group, lift-off times were faster on aligned trials than on misaligned trials, *F*(1, 29) = 5.41, *p* = .027, η^2^ = .16, and, *F*(1, 29) = 8.06, *p* = .008, η^2^ = .22, respectively.

**Fig. 5:**
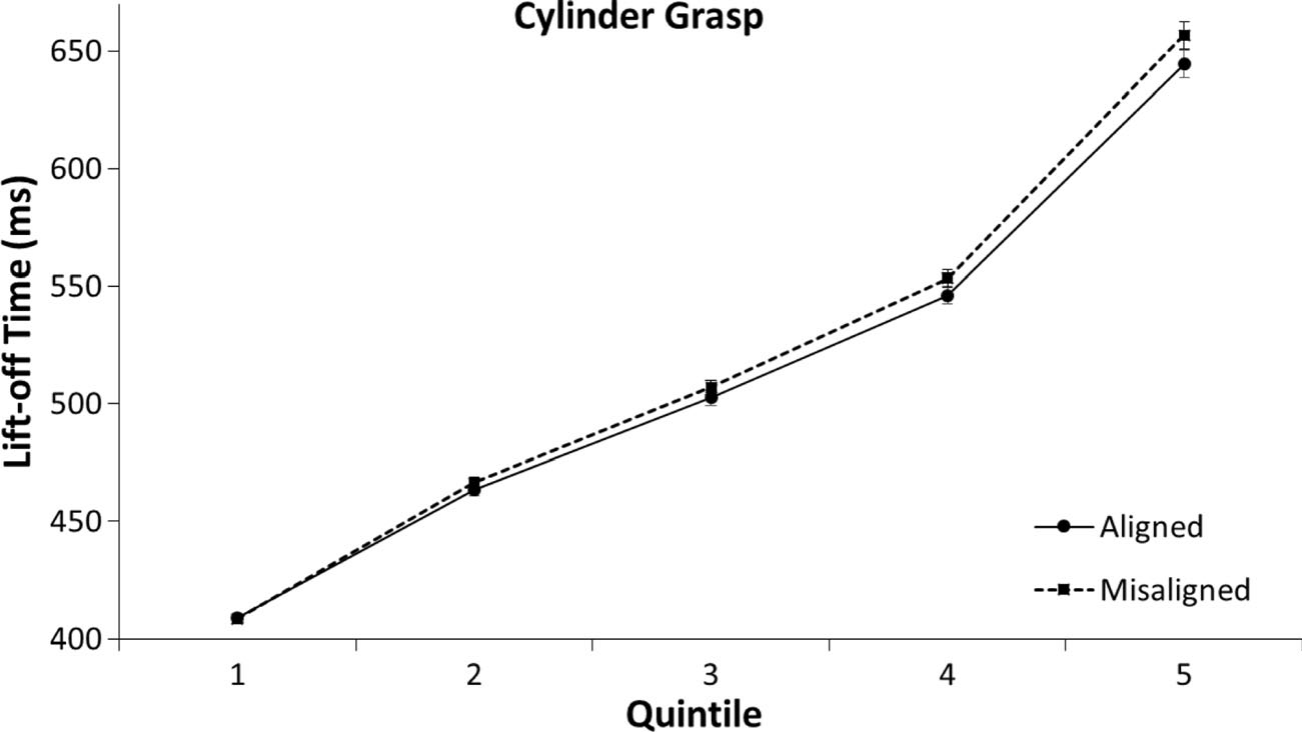
Means of individual lift-off times per quintile in the cylinder-grasp condition of Experiment 1. The error bars indicate the standard error of the mean based on the within-subjects difference between the quintiles of the aligned and misaligned conditions

**Fig. 6:**
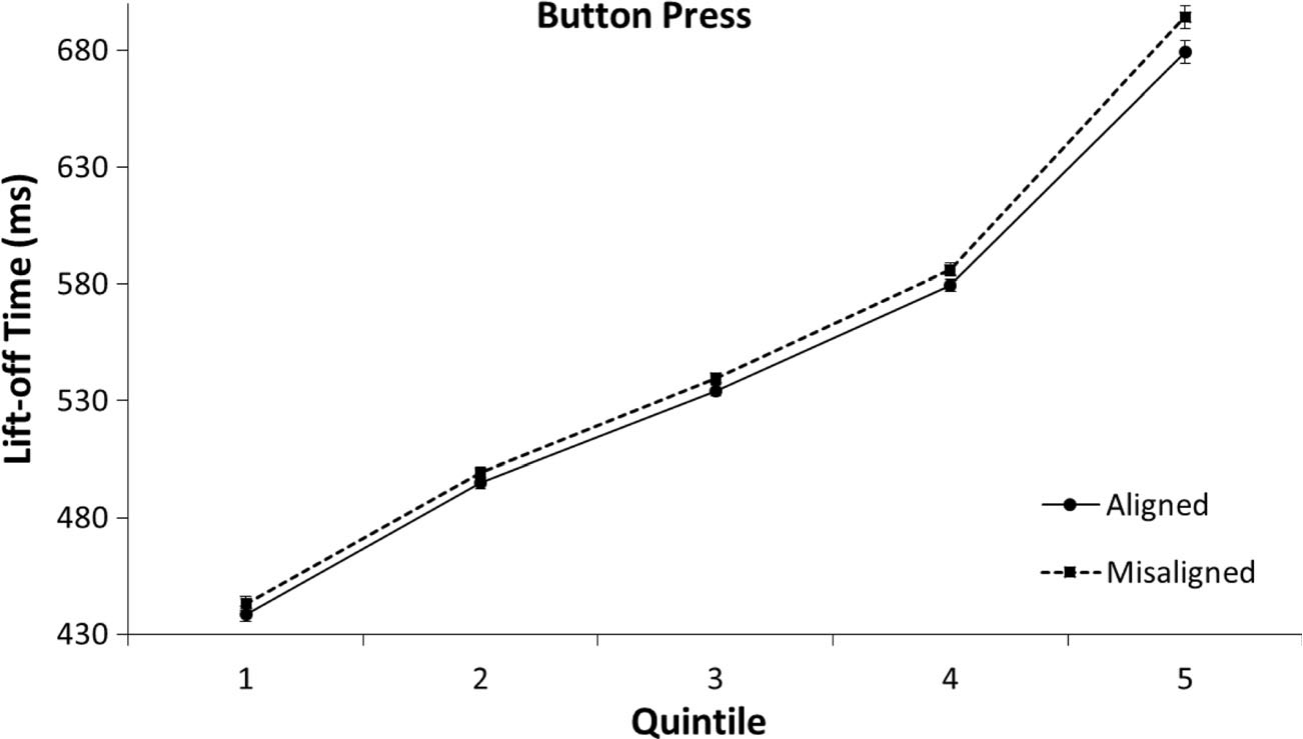
Means of individual lift-off times per quintile in the button-press condition of Experiment 1. The error bars indicate the standard error of the mean based on the within-subjects difference between the quintiles of the aligned and misaligned conditions

In an exploratory analysis of the time course of alignment effects, we calculated the mean reaction times for the first to fifth quintile of the rank-ordered RTs from the aligned and misaligned conditions (for each condition and participant separately). A 2 (aligned vs. misaligned) × 2 (cylinder grasp vs. button press) × 5 (Quintile 1 to 5) mixed ANOVA was performed on the mean lift-off times for correct trials in order to analyze this distribution. The analysis showed that lift-off times were faster on aligned trials than on misaligned trials, *F*(1, 58) = 11.30, *p* = .001, η^2^ = .16. The analysis also showed an interaction between alignment and quintile, *F*(4,58) = 6.58, *p* < .001, η^2^ = .10. No other interactions were significant (all *p*s > .7). As can be seen in Figs. 5 and 6, the mean difference in lift-off time between aligned and misaligned conditions increased in size from the first to the fifth quintile. This is reflected in the rise of the mean differences in RTs between the aligned and misaligned conditions both in the grasp group, *MD*_q1_^3^ = 0.3 ms, *t*(29) *=* 0.17, *p* = .870; *MD*_q2_ = 3.0 ms, *t*(29) *=* 1.21, *p* = .870; *MD*_q3_ = 4.5 ms, *t*(29) *=* 1.46, *p* = .154; *MD*_q4_ = 7.0 ms, *t*(29) *=* 1.97, *p* = .058; *MD*_q5_ = 12.4 ms, *t*(29) *=* 2.12, *p* = .043, and in the button-press group, *MD*_q1_ = 4.4 ms, *t*(29) *=* 1.40, *p* = .172; *MD*_q2_ = 4.3 ms, *t*(29) *=* 1.62, *p* = .116; *MD*_q3_ = 5.3 ms, *t*(29) *=* 2.22, *p* = .034; *MD*_q4_ = 6.7 ms, *t*(29) *=* 2.47, *p* = .020; *MD*_q5_ = 15.2 ms, *t*(29) *=* 3.10, *p* = .004.

#### Movement time

**Fig. 7:**
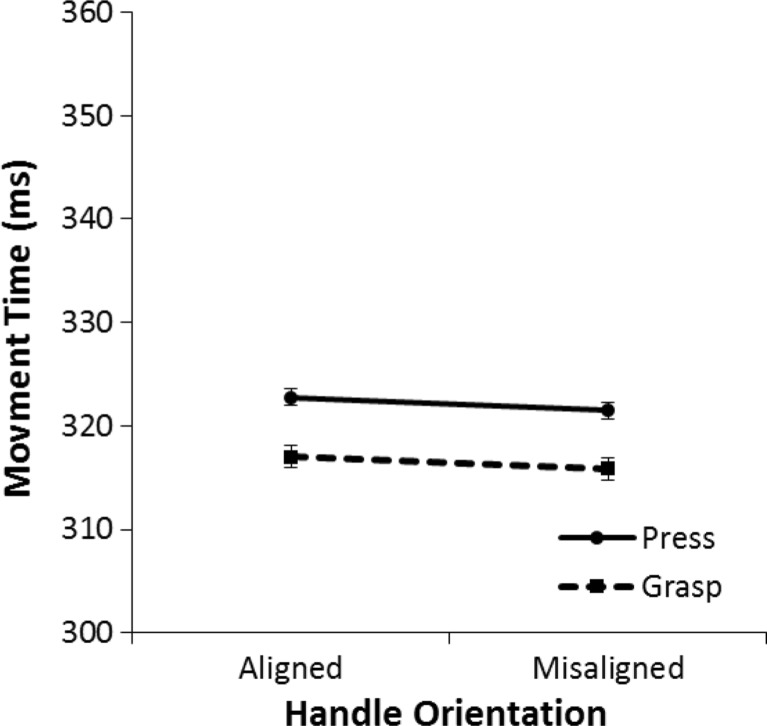
Mean movement times in Experiment 1 for the button-press group and the cylinder-grasp group. Movement time is the time it takes the hand to move from the keyboard release to the Grabbit response element. The error bars indicate the standard error of the mean based on the within-subjects difference between the aligned condition and the misaligned condition

Figure 7 displays the results for mean movement times. A 2 (aligned vs. misaligned) × 2 (cylinder grasp vs. button press) mixed ANOVA was performed on the mean movement time for correct trials. Movement times tended to be somewhat slower on aligned trials than on misaligned trials, but the effect was only marginally significant, *F*(1, 58) = 3.31, *p* = .074, η^2^ = .05. There was no difference in movement times between participants who performed a cylinder grasp response and participants who performed a button press response, *F*(1, 58) = .08, *p* = .78, η^2^ = .001. Finally, there was no interaction effect between alignment and response type, *F*(1, 58) = .01, *p* = .91, η^2^ < .001.

#### Accuracy

Table 1 displays the results for mean percentage correct trials. A 2 (aligned vs. misaligned) × 2 (cylinder grasp vs. button press) mixed ANOVA was performed on the percentage correct trials. There was no main effect of alignment, *F*(1, 58) = .71, *p* = .402, η^2^ = .01 or response type (cylinder grasp vs. button press), *F*(1, 58) = .66, *p* = .798, η^2^ < .01. In addition, there was no interaction effect between alignment and response type, *F*(1, 58) = .18, *p* = .673, η^2^ < .01.

**Table 1:** Percentage Correct Trials for Both Aligned and Misaligned Trials per Experiment and Task

Experiment	Task/Condition	Aligned		Misaligned	
		*M*	*SE*	*M*	*SE*
1	Cylinder grasp	98.8	0.2	98.6	0.3
	Button press	98.7	0.3	98.6	0.3
2	Go/no-go	99.7	0.2	99.2	0.4
	CRT	98.7	0.3	97.7	0.5
3	Go/no-go	99.6	0.2	99.3	0.2
	CRT	97.7	0.5	97.3	0.6

*Note.* CRT = left/right choice-reaction task. The CRT in Experiments 2 and 3 was identical to the cylinder grasp condition in Experiment 1

To summarize, we found an alignment effect of 7 ms on lift-off time in both the button-press and cylinder-grasp conditions. On trials where the handle of the beer mug was aligned with the correct response hand, participants responded faster than on trials where the handle of the beer mug was misaligned with the correct response hand. An exploratory analysis on the time course of the alignment effect indicated that this effect was most prominent in the slower lift-off times. Our results also showed a marginally significant main effect of response type (cylinder grasp vs. button press) on lift-off time. Participants most likely needed more time to prepare to touch the screw than to grasp the cylinder, because the screw was smaller than the cylinder.

The presence of alignment effects (of equal size) in both the button-press and cylinder-grasp conditions suggests that the alignment does not occur because of similarities between the performed response action and the grasping action afforded by the object displayed in the picture. This confirms the idea that alignment effects depend on specific task demands. The task requirement to choose between a left- or right-hand response might be the crucial factor that elicits the alignment effect. As Bub and Masson (2010) predicted, if the type of grasp is not task relevant, the alignment effect is not dependent on the specific type of grasp afforded by the pictured object. Our results are consistent with other findings of alignment effects when the response action has little similarity with grasping actions (Cho & Proctor, 2010, 2011; Phillips & Ward, 2002), and in addition indicate that response similarity does not influence the size of the alignment effect.

The object alignment effect has always been investigated in tasks where a binary left/right response had to be made. Thus, the idea that response competition is important for the presence of alignment effects or that an overlapping spatial dimension between stimulus and response is necessary for the presence of alignment effects is consistent with the literature but has never been tested directly. To investigate the importance of left/right hand selection, we removed the left/right decision from the response by using a go/no-go paradigm in Experiment 2. If the alignment effect occurs because of competition at the response level (Bub & Masson, 2010) or abstract spatial codes (Cho & Proctor, 2010), the alignment effect should disappear when the left/right decision is removed from the task. On the other hand, if the alignment effect is caused by the automatic activation of lateralized grasping responses independent of the competition between left or right limb, the alignment effect should still occur even when only one potential action is required to respond (Dixon, Goslin, & Ellis, 2012). The photograph of the beer mug would activate an automatic lateralized hand action aligned with the corresponding hand, and as a result, responses should be facilitated when the handle orientation corresponds to the response hand compared to when it does not.

## Experiment 2

In Experiment 2, participants performed two tasks: a go/no-go task and a left/right choice-reaction task (CRT). The same stimuli as in Experiment 1 were presented. Participants again responded to the color of a beer mug by grasping the cylinder on the Grabbit so that the go response resembled Bub and Masson’s (2010) grasp condition and should maximally facilitate automatic motor activation. In the go/no-go task, participants responded to one color (go trials) by grasping the cylinder with one hand and did not respond to the other color (no-go trials). On go trials, the beer mug handle could be aligned or misaligned with the response hand. The CRT was identical to the grasp condition of Experiment 1. Participants performed both tasks, but the go/no-go task always preceded the CRT. This order was used because task demands can carry over in a go/no-go task if it is preceded by a CRT (Ansorge & Wühr, 2004), presumably because earlier task demands can carry over spatial response representations to the go/no-go task. This could invoke activation of both limbs simultaneously.

### Method

#### Participants

Thirty-two psychology students at the Erasmus University Rotterdam participated in the experiment for course credit.^4^

#### Materials, apparatus, and procedure

The same stimuli as in Experiment 1 were used. The Grabbit, keyboard, and computer monitor were positioned in the same manner as in Experiment 1. Responses were also given in the same way as in Experiment 1, except that all participants responded by grasping the cylinder (i.e., there was no button-press condition).

All participants first performed the go/no-go task followed by the CRT (Ansorge & Wühr, 2004). Each task consisted of a practice phase of 20 trials followed by three blocks of critical trials of 60 trials each (180 critical trials in total). For both the go/no-go and the CRT, the practice trials were shortened to 20 trials (instead of the 40 practice trials that were used in Experiment 1) because the tasks were easily understood. Each block in the go/no-go task consisted of 30 go trials (15 aligned, 15 misaligned) and 30 no-go trials (15 with the handle of the beer mug oriented to the left and 15 with the handle oriented to the right). Each block of the CRT consisted of 30 trials that required a right-hand response (15 aligned, 15 misaligned) and 30 trials that required a left-hand response (15 aligned, 15 misaligned).

Participants were asked to respond by grasping the cylinder in response to the color of the beer mug. In the practice phase of the go/no-go task, participants were told with which hand (left or right) they had to respond and to which color they had to respond (red or blue). Participants were instructed to place the index finger of their response hand on the *v* key and to return to this key after each trial. After these instructions, participants performed 20 practice trials, where they had to respond to colored rectangles. The assignment of color to the go or the no-go condition and of color to response hand (left or right) was counterbalanced across participants so that all four combinations of color and response hand assignment were used equally often. Each trial started with the presentation of a fixation cross (*+*) for the duration of 1,500 ms. Based on the rectangle color, participants had to either respond (go trials) or do nothing (no-go trials). Feedback was displayed (*incorrect*) when participants responded during no-go trials. Each trial ended with an intertrial interval of 1,000 ms.

After these practice trials, participants were instructed to respond to the color of the beer mug, followed by three blocks of critical trials. The timing of the task was the same as in Experiment 1 (see Fig. 3), but participants only responded on go trials. After each block participants could take a self-paced break. All trials were presented in a random order for each subject.

The go/no-go task was followed by the CRT. The CRT was identical to the cylinder-grasp condition of Experiment 1.

### Results and discussion

The mean accuracy rates across participants were 99% in the go/no-go task (range = 91%–100%) and 98% in the CRT (range = 91%–100%). No participants were excluded based on accuracy. On the basis of the same outlier criteria as Experiment 1, 2.7% of the responses in the go/no-go task and 4.6% of the responses in the CRT were classified as outliers in lift-off time and movement time and excluded from the RT analyses.

#### Lift-off time

**Fig. 8:**
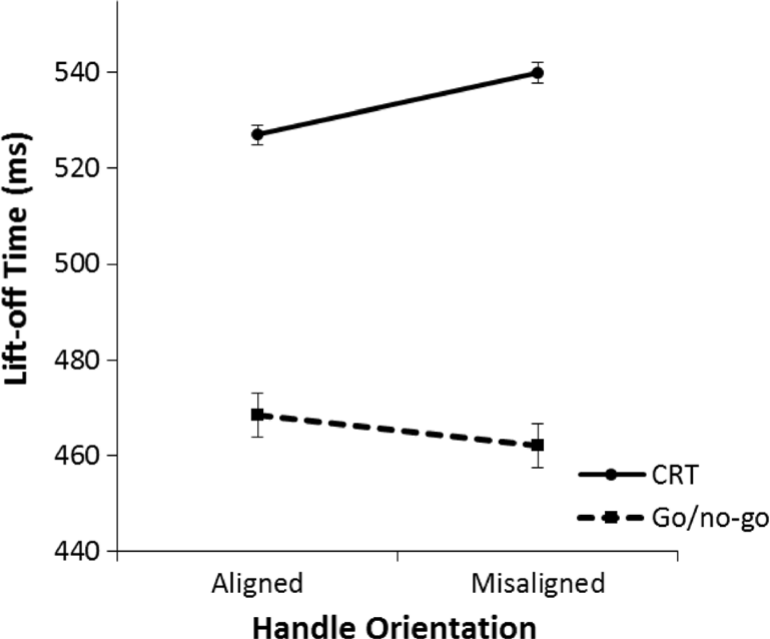
Mean lift-off times in Experiment 2 for the left/right choice-reaction task (CRT) and go/no-go task. Lift-off time is the time it takes from color onset to the beginning of the movement (release of the keyboard). The error bars indicate the standard error of the mean based on the within-subjects difference between the aligned condition and the misaligned condition

A 2 (aligned vs. misaligned) × 2 (go/no-go task vs. CRT) repeated-measures ANOVA was performed on the mean lift-off times of correct trials. The results are shown in Fig. 8. There was no overall effect of alignment, *F*(1, 31) = 1.85, *p* = .184, η^2^ = .06. Participants responded faster in the go/no-go task than in the CRT, *F*(1, 31) = 50.71, *p* < .001, η^2^ = .62. Most important, there was a significant interaction effect, *F*(1, 31) = 13.29, *p* =.001, η^2^ = .30.

Post hoc repeated-measures ANOVAs were performed to analyze alignment effects for each task separately. Contrary to the view that alignment effects are due to the automatic activation of motor actions by object pictures, participants did not respond faster on aligned trials than on misaligned trials in the go/no-go task. Rather, the ANOVA revealed that in the go/no-go task participants responded slower on aligned trials than on misaligned trials, *F*(1, 31) = 8.90, *p* = .006, η^2^ = .22. In the CRT, participants responded faster on aligned trials than on misaligned trials *F*(1, 31) = 7.99, *p* = .008, η^2^ = .21, replicating the results of Experiment 1.

As in Experiment 1, we performed an exploratory analysis of the time course of alignment effects by calculating the mean reaction times for the first to fifth quintile of the rank-ordered RTs for each condition and participant. A 2 (aligned vs. misaligned) × 2 (go/no-go task vs. CRT) × 5 (Quintile 1 to 5) repeated measures ANOVA on the mean lift-off times for correct trials showed a three-way interaction between alignment, task and quintile, *F*(4,31) = 10.73, *p* < .001, η^2^ = .26. The three-way interaction indicated that the pattern of results was different for the go/no-go task and the CRT. We therefore performed two additional ANOVAs for each task separately.

**Fig. 9:**
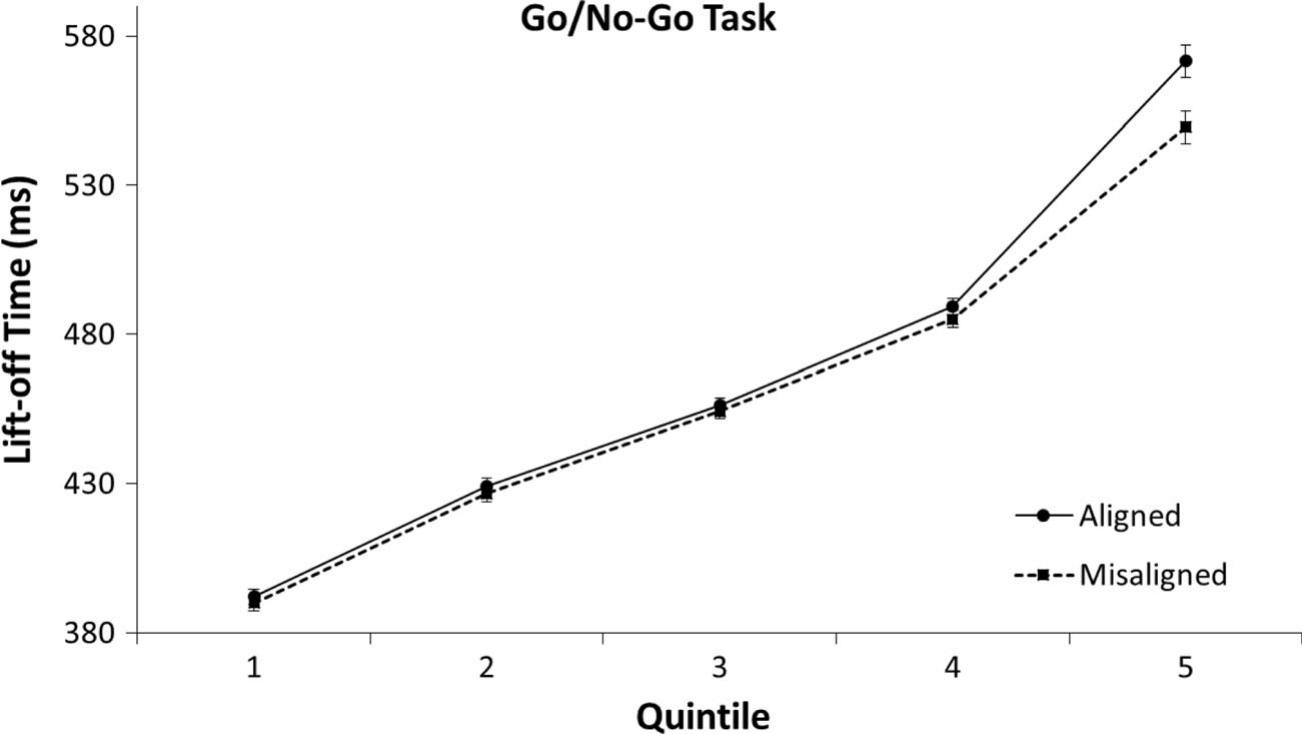
Means of individual lift-off times per quintile in the go/no-go task of Experiment 2. The error bars indicate the standard error of the mean based on the within-subjects difference between the quintiles of the aligned and misaligned conditions

The 2 (aligned vs. misaligned) × 5 (Quintile 1 to 5) repeated-measures ANOVA on lift-off times for the go/no-go task revealed an interaction between alignment and quintile, *F*(4,31) = 9.72, *p* < .001, η^2^ = .24. As can be seen in Fig. 9 a negative alignment effect (slower responses for aligned than for misaligned trials) was only apparent in the fifth (slowest) quintile in the go/no-go task, *MD*_q1_ = -2.1 ms, *t*(31) *=* .85, *p* = .403; *MD*_q2_ = -2.6 ms, *t*(31) = 1.01, *p* = .318; *MD*_q3_ = -2.0 ms, *t*(31) *=* .77, *p* = .447; *MD*_q4_ = -4.3 ms, *t*(31) *=* 1.53, *p* = .137; *MD*_q5_ = -22.1 ms, *t*(31) *=* 4.03, *p* < .001.

**Fig. 10:**
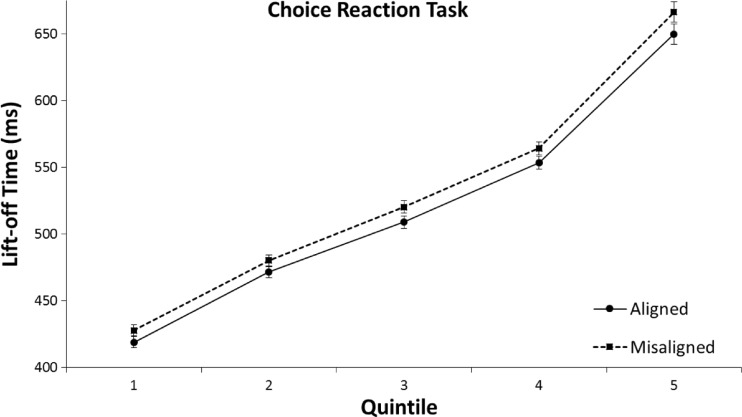
Means of individual lift-off times per quintile in the left/right choice-reaction task (CRT) of Experiment 2. The error bars indicate the standard error of the mean based on the within-subjects difference between the quintiles of the aligned and misaligned conditions

The 2 (aligned vs. misaligned) × 5 (Quintile 1 to 5) repeated-measures ANOVA on lift-off times for the CRT did not show an interaction between alignment and quintile, *F*(4, 31) = 1.26, *p* = .270, η^2^ = .04. Thus, as shown in Fig. 10, the mean RT difference between aligned and misaligned trials was approximately equal for all quintiles.

#### Movement time

**Fig. 11:**
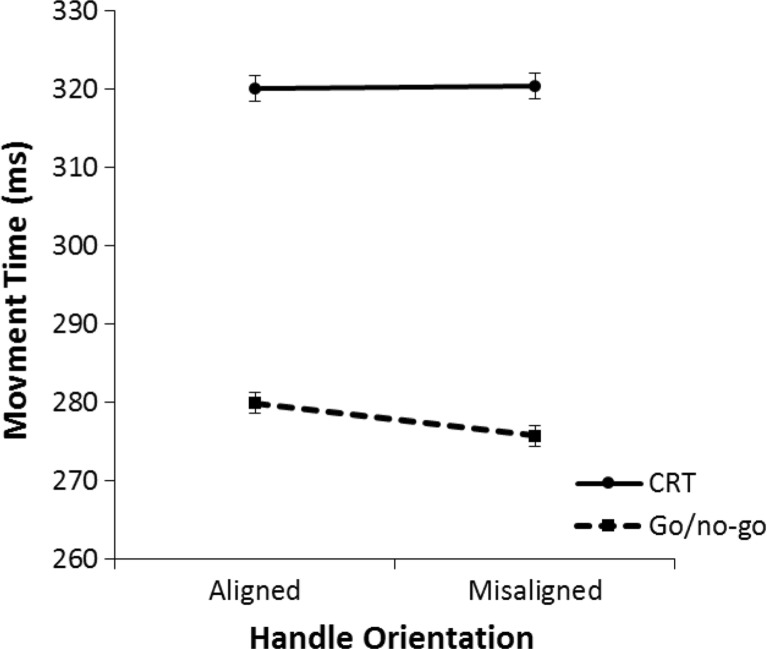
Mean movement times in Experiment 2 for the left/right choice-reaction task (CRT) and go/no-go task. Movement time is the time it takes the hand to move from the keyboard release to the Grabbit response element. The error bars indicate the standard error of the mean based on the within-subjects difference between the aligned condition and the misaligned condition

A 2 (aligned vs. misaligned) × 2 (go/no-go vs. CRT) repeated-measures ANOVA was performed on the mean movement time for all correct trials. Figure 11 displays the mean movement times for each condition. There was a marginally significant effect of alignment on movement time; movement time tended to be slower on aligned trials than on misaligned trials, *F*(1, 31) = 3.28, *p* = .080, η^2^ = .10. Participants responded faster in the go/no-go task than in the CRT, *F*(1, 31) = 21.05, *p* < .001, η^2^ = .40. There was a marginally significant interaction effect, *F*(1, 31) = 4.02, *p* = .054, η^2^ = .12.

Post hoc repeated-measures ANOVAs were performed to analyze alignment effects for each task separately. These analyses revealed that in the go/no-go task participants responded slower on aligned trials than on misaligned trials, *F*(1, 31) = 6.23, *p* = .018, η^2^ = .17. No effect of alignment was found in the CRT, *F*(1, 31) = 0.06, *p* = .809, η^2^ < .01. Similar to the results in lift-off time, an exploratory analysis was performed on the time course of alignment effects. The 2 (aligned vs. misaligned) × 2 (go/no-go task vs. CRT) × 5 (Quintile 1 to 5) repeated-measures ANOVA did not reveal any significant interactions with quintiles (all *p*s > .22).

#### Accuracy

Table 1 displays the results for mean percentage correct trials. A 2 (aligned vs. misaligned) × 2 (go/no-go task vs. CRT) repeated measures ANOVA was performed on the percentage correct trials. Participants had a higher percentage correct for aligned trials than misaligned trials, *F*(1, 31) = 9.23, *p* = .005, η^2^ = .23. Participants had a higher percentage correct for the go/no-go task than CRT, *F*(1, 31) = 6.20, *p* = .018, η^2^ = .17. There was no interaction effect between handle orientation and task, *F*(1, 31) = .62, *p* = .439, η^2^ = .02.

To summarize, the CRT in Experiment 2 replicated the findings of Experiment 1. The mean alignment effect in the CRT of Experiment 2 was about 13 ms. However, there was a negative alignment effect in the go/no-go task of about 6 ms. This effect was most prominent in the slowest lift-off times. In the go/no-go task, participants responded faster when the handle was misaligned with the response hand while they responded faster when the handle was aligned with the response hand in the CRT. It is unclear why in the go/no-go task the alignment effect was reversed. Negative alignment effects have occasionally been reported (e.g., Kostov & Janyan, 2015; Yu et al., 2014), but these studies did not use a go/no-go task. Because we did not find a negative alignment effect in other go/no-go experiments (see footnote 4 and Experiment 3), we will refrain from further discussing this finding.

In Experiments 1 and 2, we used a color-decision task so that object shape was irrelevant to the response. According to Tipper et al. (2006), in order to evoke automatic lateralized motor responses, the shape of the object needs to be attended (see also Symes, Ellis, & Tucker, 2005; but see Cho & Proctor, 2010, 2011, 2013). It is therefore possible that automatic motor response evoked by the picture of the beer mug was not sufficiently activated in the previous two experiments. To further investigate the role of automatic lateralized hand actions in the alignment effect, we made object shape relevant to the response in a manner similar to the study of Tucker and Ellis (1998) to be certain that the results we found in Experiments 1 and 2 were not due to the lack of attention to object shape.

## Experiment 3

In Experiment 3, participants again performed a go/no-go task and a left/right choice-reaction task (CRT) in response to the beer mug. This time they did not respond to the color of the beer mug but to their upright or inverted orientation. If automatic lateralized motor responses only appear when the object shape is task relevant, we expect to find alignment effects in the go/no-go task. If, however, handle alignment only facilitates responses due to competing action representations or abstract spatial codes, we expect no alignment effects in the go/no-go task but do expect alignment effects in the CRT. Participants again responded by grasping the cylinder on the Grabbit.

### Method

#### Participants

Thirty-two psychology students of the Erasmus University Rotterdam participated in the experiment for course credit. Two additional participants were tested but not included in the analyses; one was excluded from the analyses because of hardware malfunctions. One was excluded from the analysis because of a failure to perform the go/no-go task correctly.

#### Materials and apparatus

The same grayscale photograph of the beer mug that was used in Experiments 1 and 2 was used in Experiment 3. The beer mug was either displayed upright or inverted with the handle oriented to the left or the right. Responses were given in the same way as in Experiment 2.

#### Procedure

The procedure resembled that of Experiment 2 with slight modifications. The beer mug was shown either upright or inverted with the handle on the right or left, which resulted in four different stimuli. There was no cue delay because the beer mug did not change color. In the practice phase of the go/no-go task participants were instructed which hand to use and to which orientation they had to respond (upright or inverted). The assignment of mug orientation (upright or inverted) to response hand (left or right) was counterbalanced between participants, resulting in four counterbalanced versions. Practice trials also consisted of an upright or inverted beer mug. In the beginning of the practice phase of the CRT, participants were told which hand they were supposed to use in response to the orientation of the beer mug (upright or inverted).

### Results and discussion

Participants had a mean accuracy of 98% in the go/no-go task (range = 77%, a single participant performed poorly, to 100% correct responses) and had a mean accuracy of 96% in the CRT (range = 89%–99%). No participants were excluded based on accuracy. Participants responded slower in Experiment 3 than in Experiments 1 and 2. After visual inspection of the overall RT distribution we increased the cutoff rate for outliers in lift-off time to 1,500 ms (compared to 1,000 ms in Experiments 1 and 2). All other cutoff rates remained the same as in Experiment 1 and 2. This resulted in 0.4% of the responses in the go/no-go task and 2.5% of the responses in the CRT to be classified as outliers and excluded from the RT analyses.

#### Lift-off time

**Fig. 12:**
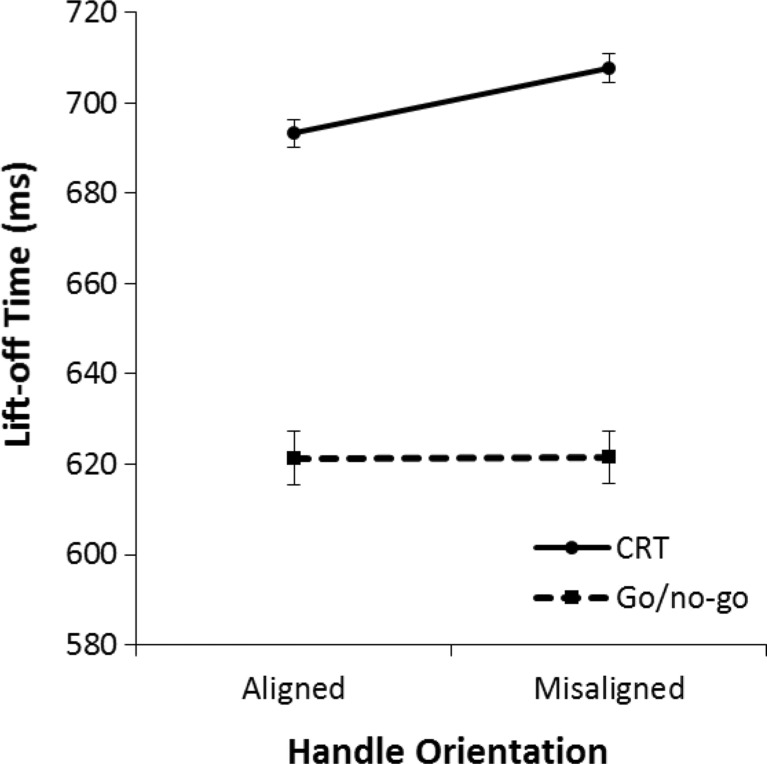
Mean lift-off times in Experiment 3 for the left/right choice-reaction task (CRT) and go/no-go task. Lift-off time is the time it takes from the onset of stimulus presentation until the start of the movement (release of the keyboard). The error bars indicate the standard error of the mean based on the within-subjects difference between the aligned condition and the misaligned condition

To examine the effects on lift-off time, a 2 (aligned vs. misaligned) × 2 (go/no-go task vs. CRT) repeated-measures ANOVA was performed on the mean lift-off reaction time (RT) of all correct trials. Figure 12 illustrates that mean lift-off times were faster on aligned trials than on misaligned trials, *F*(1, 31) = 5.24, *p* = .029, η^2^ = .15. Also, participants responded faster in the go/no-go task than in the CRT, *F*(1, 31) = 25.09, *p* < .001, η^2^ = .45. More important, there was a significant interaction effect, *F*(1, 31) = 4.25, *p* =.048, η^2^ = .12.

Post hoc repeated-measures ANOVAs were performed to analyze the effects for each task separately. These analyses revealed that in the go/no-go task there was no effect of alignment, *F*(1, 31) = 0.01, *p* = .932, η^2^ < .001. In the CRT, lift-off times were faster on trials where the handle of the beer mug was aligned with the correct response hand than on trials where the handle of the beer mug was misaligned with the correct response hand, *F*(1, 31) = 6.04, *p* = .020, η^2^ = .16.

An exploratory analysis of the RT distribution was performed by calculating the mean reaction times for the first to fifth quintile of the rank-ordered RTs for each condition and participant. A 2 (aligned vs. misaligned) × 2 (go/no-go task vs. CRT) × 5 (quintile) repeated-measures ANOVA was performed on the mean lift-off times for correct trials in order to analyze this distribution. The analysis showed a three-way interaction between alignment, task, and quintile, *F*(4, 31) = 3.64, *p* = .008, η^2^ = .11. The three-way interaction indicated that the pattern of results was different for the go/no-go task and the CRT. We therefore performed two additional ANOVAs for each task separately.

**Fig. 13:**
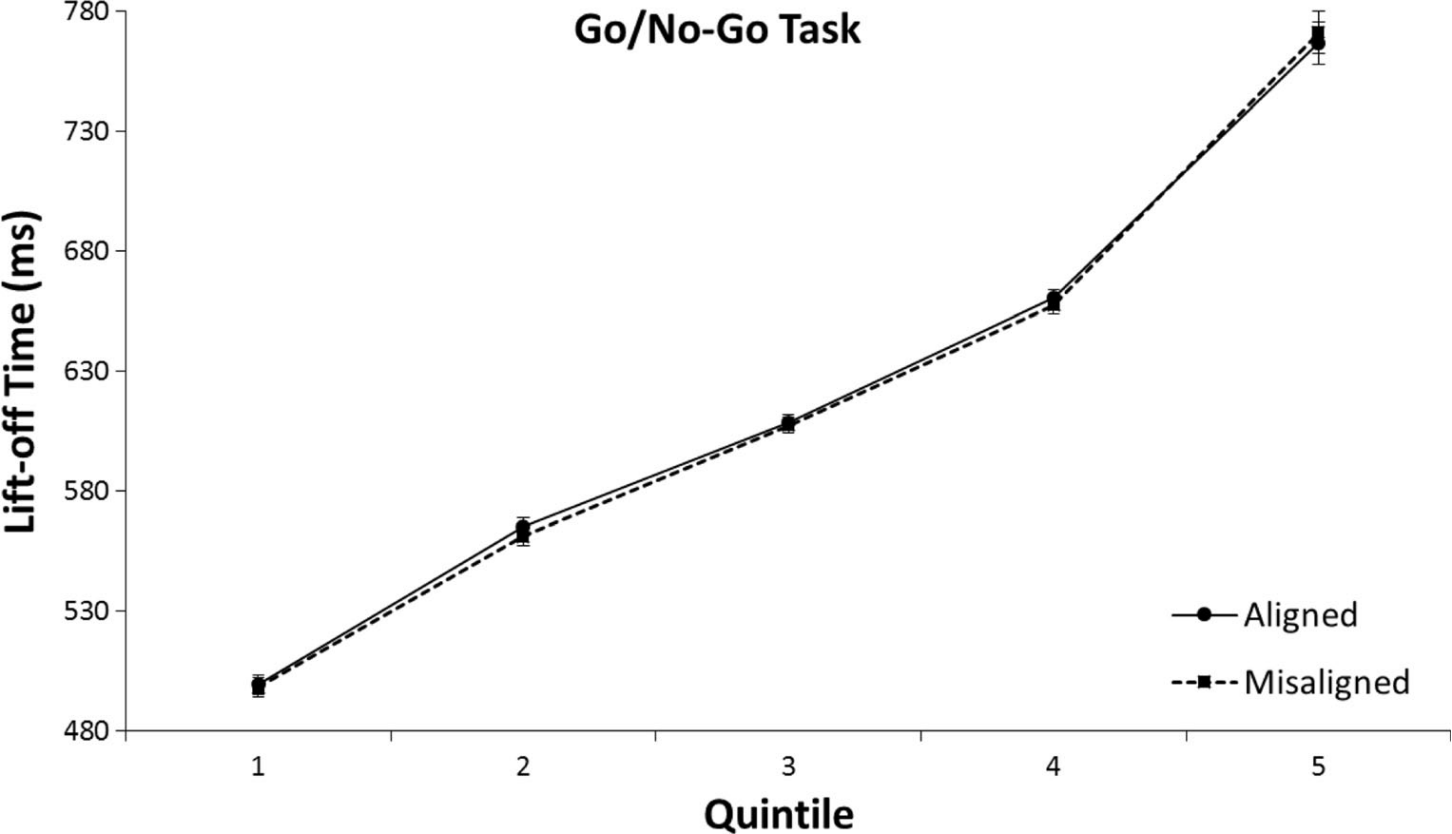
Means of individual lift-off times per quintile in the go/no-go task of Experiment 3. The error bars indicate the standard error of the mean based on the within-subjects difference between the quintiles of the aligned and misaligned conditions

The 2 (aligned vs. misaligned) × 5 (Quintile 1 to 5) repeated-measures ANOVA on lift-off times for the go/no-go task did not show an interaction between alignment and quintile, *F*(4, 31) = 0.56, *p* = .695, η^2^ = .02 (see Fig. 13).

**Fig. 14:**
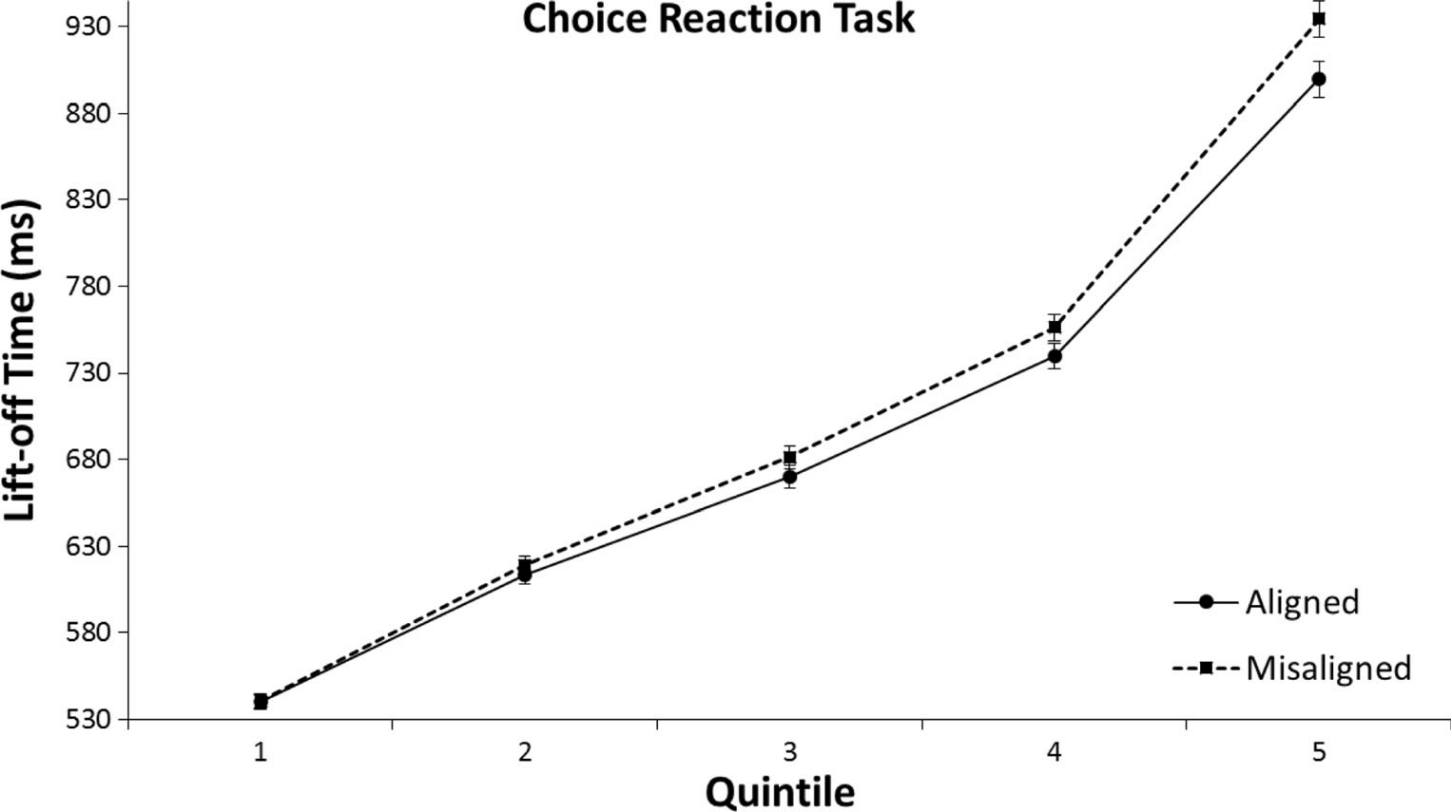
Means of individual lift-off times per quintile in the left/right choice-reaction task (CRT) of Experiment 3. The error bars indicate the standard error of the mean based on the within-subjects difference between the quintiles of the aligned and misaligned conditions

The 2 (aligned vs. misaligned) × 5 (Quintile 1 to 5) repeated-measures ANOVA on lift-off times for the CRT showed an marginally significant interaction between alignment and quintile, *F*(4, 31) = 2.24, *p* = .069, η^2^ = .07. As can be seen in Fig. 14, in the CRT the difference between aligned and misaligned tended to increase in size from the faster to the slower responses. This was reflected in the rise of mean RT differences between aligned and misaligned trials, *MD*_q1_ = 0.3 ms, *t*(31) *=* .07, *p* = .945; *MD*_q2_ = 6.2 ms, *t*(31) *=* 1.21, *p* = .235; *MD*_q3_ = 11.6 ms, *t*(31) *=* 1.74, *p* = .092; *MD*_q4_ = 16.6 ms, *t*(31) *=* 2.18, *p* = .037; *MD*_q5_ = 34.8 ms, *t*(31) *=* 3.34, *p* = .002.

#### Movement time

**Fig. 15:**
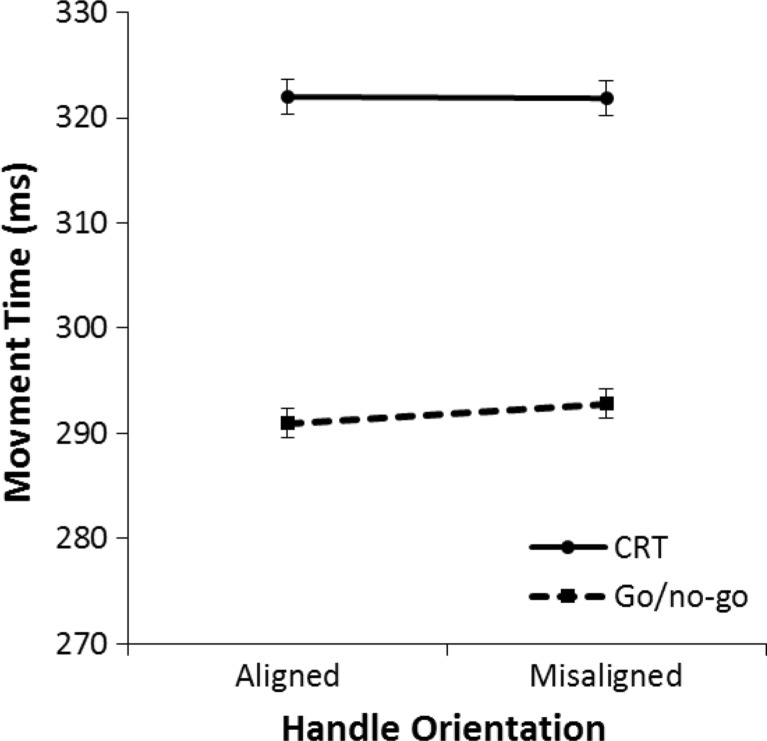
Mean movement times in Experiment 3 for the left/right choice-reaction task (CRT) and go/no-go task. Movement time is the time it takes the hand to move from the keyboard release to the Grabbit response element. The error bars indicate the standard error of the mean based on the within-subjects difference between the aligned condition and the misaligned condition

To examine the effects on movement time a 2 (aligned vs. misaligned) × 2 (go/no-go vs. CRT) repeated-measures ANOVA was performed on the mean movement time of all correct trials. Figure 15 illustrates the effects found in this analysis. There was no effect of alignment on movement time, *F*(1, 31) = .53, *p* = .473, η^2^ = .02. Participants responded faster in the go/no-go task than in the CRT, *F*(1, 31) = 13.82, *p* = .001, η^2^ = .31. There was no significant interaction effect, *F*(1, 31) = .96, *p* = .336, η^2^ = .03.

#### Accuracy

Table 1 displays the results for mean percentage correct trials. A 2 (aligned vs. misaligned) × 2 (go/no-go vs. CRT) repeated measures ANOVA was performed on the percentage correct trials. Participants did not differ in percentage correct between aligned and misaligned trials, *F*(1, 31) = .2.01, *p* = .166, η^2^ = .06. Participants had a higher percentage correct for the go/no-go task than the CRT, *F*(1, 31) = 21.53, *p* < .001, η^2^ = .41. There was no interaction effect between alignment and task, *F*(1, 31) = .03, *p* = .860, η^2^ < .01.

The results of Experiment 3 largely replicated those of Experiment 2, and the CRT also replicated findings of Experiment 1. In contrast to Experiment 2, where the alignment effect seemed to be reversed in the go/no-go task, in Experiment 3 no alignment effect was found in the go/no-go task. The results of Experiment 3 show no indication of automatically activated lateralized motor activation when object shape is relevant to the response.

## General discussion

The purpose of our study was to examine if the alignment effects in response to depicted graspable objects are due to automatically activated motor programs. In Experiments 1 and 2, participants responded to the color (red or blue) of a beer mug displayed on a computer screen. The handle of the beer mug was oriented to either the right or the left. Our study replicated previous findings (Bub & Masson, 2010) that an object’s graspable part elicited faster reaction times if the side of the graspable part and the response hand were aligned than if they were misaligned. More specifically, we found that this effect occurred not only when participants were asked to respond by grasping a cylinder but also when they were asked to respond by touching a screw with their index finger after a reaching movement, suggesting that overlap in the grasping action associated with the beer mug and the executed response did not play a role. These alignment effects were found in the time it took from stimulus onset to the initiation of the movement (lift-off time) but not in movement time. Both findings suggest that the alignment effect occurred during response hand selection rather than during response execution. In Experiment 2 alignment effects were found in the left/right choice-reaction task (CRT) similar to Experiment 1, but in the go/no-go task the alignment effect was reversed. Finally, in Experiment 3 we tried to increase the potential activation of motor actions by making the shape of the beer mug relevant to the response. Participants responded to the upright or inverted orientation of the beer mug. This task has more reliably shown alignment effects than the color decision task (Tipper et al., 2006; Tucker & Ellis, 1998; but see Yu et al., 2014). An alignment effect was present in the CRT but not in the go/no-go task. Thus, the alignment effect disappeared when response selection between competing left and right responses was no longer required. Our findings thus show that lateralized motor actions toward the object handle are not activated automatically by viewing an object but may depend on competition at the response level due to task demands or to abstract spatial codes.

Earlier research into alignment effects elicited by the handle orientation of objects used paradigms that asked participants to respond with left/right responses (e.g., Bub & Masson 2010; Cho & Proctor, 2010, 2011, 2013; Goslin et al., 2012; Iani et al., 2011; Lien et al., 2014; Pellicano et al., 2010; Phillips & Ward, 2002; Song et al., 2014; Tipper et al., 2006; Tucker & Ellis, 1998). In Experiment 1, we manipulated the response action and its similarity to the grip associated with the presented object and found no effect of this manipulation. In Experiments 2 and 3, we manipulated the availability of left/right response representations by including a go/no-go condition.

The alignment effects found in the CRTs are most likely due to task-induced competition, either between the left and right limb at the response level (Bub & Masson, 2010) or between abstract spatial codes (e.g., Cho & Proctor, 2010). The presence of two response locations might focus attention on the stimulus’ left/right orientation, increasing the salience of the left or right side of the object. Both views predict an alignment effect only when the task requires a response decision (as in Experiment 1 and the CRT in Experiments 2 and 3) and predict that the alignment effect does not occur when the left/right dimension is removed from the response (as in the go/no-go tasks of Experiments 2 and 3).

It is worth pointing out that alignment effects can be absent even when a left/right dimension is present. For example, Yu et al. (2014) did not find alignment effects when participants were instructed to make a movement toward a left or right button in response to manmade or natural objects (Experiment 1c). These results seem to conflict with our current findings. There are, however, a number of important differences between our task and the one performed by Yu et al. First, in our task participants were instructed to hold down two keys, one with their left hand and one with their right hand, after which they moved one of their hands in response to the color of a depicted beer mug. In the experiment performed by Yu et al. participants held down the spacebar with their dominant hand and were instructed to move and press a button located to the left or right. In our task, the response dimension might have been more salient because participants needed to decide which hand they needed to respond with, while the task by Yu et al.’s participants needed to decide if they had to move their hand leftward or rightward. Second, Yu et al. had participants respond to an array of different man-made objects, while we only showed a single beer mug in our experiment, similar to the procedure used by Bub and Masson (2010). Furthermore, Yu et al. had participants judge if an object was man-made or natural, while we had participants respond to color or upright/inverted orientation. When participants have to decide if an object is man-made, they might focus on a specific part of an object—for example, the drill bit of a drill is more important to recognize what it is compared to the handle. This might have caused attention to be directed away from the handle and toward distinguishing parts of an object. In other words, stimulus set, response characteristics, and task demands were all different between Yu et al.’s experiments and those of us and Bub and Masson. Stimulus set and task demands have shown to influence the alignment effect in response to handled objects (e.g. Lien, et al., 2014; Song et al., 2014) and might therefore have caused these different findings.

Alignment effects are usually accompanied by task-specific attributes. For example, Tucker and Ellis (1998) instructed participants to imagine grasping the object that was shown. Yu et al. (2014) showed that finding alignment effects for key-press responses might crucially depend on this instruction. Possibly, the instruction to imagine grasping the object draws attention toward the handle, making it more salient. This sensitivity to instruction also argues against an automatic activation account of the alignment effect. Because Tucker and Ellis (1998) instructed participants to imagine grasping the object that was shown, it is likely that this would have influenced their findings. Anderson et al. (2002) presented scissors with the handles aligned to the left or right or clocks with their hands turned to the left or right. An alignment effect was found with both these objects, even though a clock hand does not afford grasping. The effect was more likely caused by an attention bias toward different parts of an asymmetrical object. Attentional bias may be increased toward objects handles when participants perform a reaching action (Bub & Masson, 2010) or are instructed to imagine grasping the object. This may increase the handle salience, which causes the alignment effect. Earlier studies have also shown that the alignment effect occurs in a color-decision task with simple left and right button presses when the handle protrudes to the left or to the right while keeping the body of the object centered (Cho & Proctor, 2011). The alignment effect does not seem to occur with simple button presses when the whole object is centered (Bub & Masson, 2010, Experiment 2). Again, this difference is most likely caused by a difference in handle salience. When the handle clearly protrudes to the left or right side, the handle becomes a more salient property of the object. When the whole object is centered, the handle is a less salient property of the object, since it no longer clearly protrudes to the left or right side.

Yu et al. (2014) argued that object shape might prime action features related to grip more strongly and more automatically than action features related to location, possibly because grip is an invariant object but location is not. Several studies have shown that the particular grip that would be used to grasp an object is activated by a picture or the name of the object (Bub & Masson, 2012; Bub, Masson, & Cree, 2008; Masson, Bub, & Warren, 2008; Till, Masson, Bub, & Driessen, 2014; Tucker & Ellis, 2001, 2004). For example, a pinch response is facilitated by *grape* compared to *apple*, whereas a power grasp is facilitated by *apple* compared to *grape.* Thus, object shape may not automatically facilitate a specific hand but might still automatically facilitate the specific type of grasp. Therefore, our results should not be taken to indicate that no motor actions are activated at all by object pictures. However, that the alignment effect in Experiment 1 was not influenced by grip congruency suggests that if grasping actions are activated automatically they do not seem to be hand specific. Insofar that both hands might generate specific motor actions at the same time in accordance with the grip, this would not result in an alignment effect.

We would like to point out that alignment effects discussed in this paper pertain to alignment effects that are caused by the horizontal spatial dimension of a handled object. We believe these effects might be caused by competition at the response level and are less influenced by automatic motor programs when passively viewing an object. Other kinds of alignment effects might also exists. For example, Bub and Masson (2010) found clear differences when a response was aligned with a grasp with the same orientation (horizontal or vertical) in response to objects that afforded either a horizontal or vertical power grasp. More recently, Till et al. (2014) have shown that the specific hand movement changes midflight to accommodate differences in affordances of pictured objects. As mentioned before, changes in task demands may cause changes in activated motor responses.

To conclude, our findings are problematic for the view that left/right alignment effects in response to pictures of graspable objects are due to automatic activation of a grasping response. We found that alignment effects were not influenced by the similarity between the grasp afforded by the depicted object and the response action. We also found that alignment of the object handle and response hand did not facilitate responding when we removed the left/right dimension from the response by using a go/no-go task. Our results lead us to question the usefulness of CRTs to provide evidence for automatic activation of a motor response to graspable objects. We conclude that the alignment effects, found in tasks that incorporate an overlap in stimulus and response dimensions, are most likely caused by task-induced competition between two response representations.

## Footnotes

1 Some researchers use the term *affordance* to refer to this idea. This is not a neutral term, however, because it implies that the effects are caused by the affordance of the pictured object, whereas other explanations exist. In addition, some researchers object to the use of affordances for pictured objects because, according to Gibson (1979), only real objects have affordances. Therefore, an object picture does not have the same affordances a real object has. Nevertheless, the term *affordance* is widely used in combination with lateralized responses to pictures of objects with graspable parts. We will, however, use the more neutral wording, namely *activation of lateralized response*.

2 In their Experiment 4, Bub and Masson (2010) made the orientation of the response task relevant, and in that case they found that correspondence in orientation of the stimulus and response did affect RTs.

3 *MD*_q1_ refers to the mean difference (*MD*) between the aligned and misaligned condition for the first quintile. The subscript indicates the quintile.

4 Experiment 2 was performed on two previous occasions. The first time we lost movement time data due to a programming error. In this experiment, there was a significant interaction effect between alignment and task in lift-off times, *F*(1, 31) = 8.26, *p* =.007, η^2^ = .21. Post hoc repeated measures ANOVAs were performed to analyze alignment effects for each task separately. In the go/no-go task, there was no effect of handle alignment, *F*(1, 31) = .21, *p* = .651, η^2^ < .01. In the choice-reaction task, participants responded faster on aligned trials than on misaligned trials *F*(1, 31) = 9.32, *p* = .005, η^2^ = .23. The second time we accidentally enlisted participants who previously participated in a similar study, where they were asked to grasp a response device with their left or right hand in response to pictures of objects. According to Ansorge and Wühr (2004), this could influence the response mapping in the go/no-go task. As expected, we did not find an interaction effect for these data. Because of the potential contamination of the earlier left/right choice-reaction task, we decided to discard this second try and to perform the experiment a third time (as it is reported in this paper) to prevent such carryover effects.
